# A Comparative Multi-Frequency EPR Study of Dipolar Interaction in Tetra-Heme Cytochromes

**DOI:** 10.3390/ijms241612713

**Published:** 2023-08-12

**Authors:** Wilfred R. Hagen, Ricardo O. Louro

**Affiliations:** 1 Department of Biotechnology, Delft University of Technology, Building 58, Van der Maasweg 9, 2629 HZ Delft, The Netherlands; 2 Instituto de Tecnologia Química e Biológica António Xavier (ITQB-NOVA), Universidade Nova de Lisboa, 2780-157 Oeiras, Portugal

**Keywords:** EPR, broadband, dipolar interaction, cytochrome, tetraheme, heme orientation, *Shewanella*, *Desulfovibrio*

## Abstract

Distances between Fe ions in multiheme cytochromes are sufficiently short to make the intramolecular dipole-dipole interaction between hemes probable. In the analysis of EPR data from cytochromes, this interaction has thus far been ignored under the assumption that spectra are the simple sum of non-interacting components. Here, we use a recently developed low-frequency broadband EPR spectrometer to establish the extent of dipolar interaction in the example cytochromes, characterize its spectral signatures, and identify present limitations in the analysis. Broadband EPR spectra of *Shewanella oneidensis* MR-1 small tetraheme cytochrome (STC) have been collected over the frequency range of 0.45 to 13.11 GHz, and they have been compared to similar data from *Desulfovibrio vulgaris* Hildenborough cytochrome *c*_3_. The two cases are representative examples of two very different heme topologies and corresponding electron-transfer properties in tetraheme proteins. While in cytochrome *c_3_*, the six Fe-Fe distances can be sorted into two well-separated groups, those in STC are diffuse. Since the onset of dipolar interaction between Fe-Fe pairs is already observed in the X-band, the *g* values are determined in the simulation of the 13.11 GHz spectrum. Low-frequency spectra are analyzed with the inclusion of dipolar interaction based on available structural data on mutual distances and orientations between all hemes. In this procedure, all 24 possible assignments of individual heme spectra to heme topologies are sampled. The 24 configurations can be reduced to a few, but inspection falls short of a unique assignment, due to a remaining lack of understanding of the fine details of these complex spectra. In general, the EPR analysis suggests the four-heme system in *c*_3_ to be more rigid than that in STC, which is proposed to be related to different physiological roles in electron transfer.

## 1. Introduction

Intramolecular dipolar interaction in multi-center metalloproteins is a likely event with center-to-center distances of the order of 10 Å (e.g., [1,2]). Its manifestation in EPR spectroscopy is typically difficult to analyze when data are only available at a single microwave frequency because their contribution to the spectra cannot be unequivocally disentangled from other interactions, such as *g* strain or unresolved hyperfine splittings. Occasionally, data taken with spectrometers running at different frequencies have been scrutinized, for example, for a Mo^5+^-[2Fe-2S]^1+^ system [3]. An analysis of heme-heme interactions in hemoproteins was never attempted until our recent work [4], which became possible by the construction of a novel type of EPR spectrometer that can be tuned to a large number of microwave frequencies over a very broad range of 0.1–18 GHz [4,5,6,7,8]. The present work is a systematic extension of our previous study [4], aiming at identifying what is now possible in terms of the analysis of dipolar interactions in multiheme proteins and what remaining shortcomings should be addressed in the future.

Extracytoplasmic cytochromes that function in electron transfer come in a large variety of multiplicity, ranging from proteins with a single heme to proteins with many dozens of hemes [9,10,11]. The presence of multiple hemes in a single protein obviously increases the distance over which reducing equivalents can be transported, even to extracellular space. However, it also allows for inter-heme interaction of electronic nature (redox interaction), chemical nature (Bohr interaction), and magnetic nature (dipolar and exchange interaction). The time span of fundamental studies on these interactions now exceeds half a century without showing any sign of retardation, thus attesting to the incompleteness of our knowledge on the subject. Small tetraheme cytochromes have acted as the prime models on practical grounds due to their stability and abundance in many bacterial species; and on theoretical grounds, due to their relatively limited complexity.

Initially, the main tool for the studies on the interaction in tetraheme cytochromes was EPR spectroscopy [12], but its subsequent use (e.g., [13,14,15,16]) appears to have become somewhat idle over the last two decades. Other tools gained preference, notably direct electrochemistry [17,18,19,20] and paramagnetic NMR spectroscopy [21,22,23,24], with a structural frame of reference provided by high-resolution protein crystallography [25,26,27,28]. Recently, the EPR angle gained new impetus in a low-frequency broadband EPR study on the dipolar interaction in cytochromes [4], and the present paper is the next step and generalization of that work.

A broadband EPR spectrometer is an instrument, in contrast to the wide-spread, conventional single-frequency spectrometers, that can be tuned to many microwave frequencies over multiple octaves. One of us has recently described such an instrument for frequencies below and around the standard X-band, approximately in the range of 0.1–18 GHz. The key novelty of this approach is to replace the single-mode cavity with a multi-mode resonator based on strip line technology [4,5,6,7,8]. Application of the spectrometer to the study of intermolecular dipolar broadening in a monoheme cytochrome and intramolecular dipolar broadening in a tetraheme cytochrome led to an unexpected result: in the tetraheme cytochrome, dipolar interaction was detectable up to X-band frequency [4]. This implies that, for this protein, X-band EPR data cannot be simply analyzed under the premise of four non-interacting hemes, although that had been the assumption in all previous EPR literature on tetraheme proteins. If the implication would be generally valid, then the complete EPR literature on tetraheme model proteins would have to be re-evaluated.

**Figure 1:**
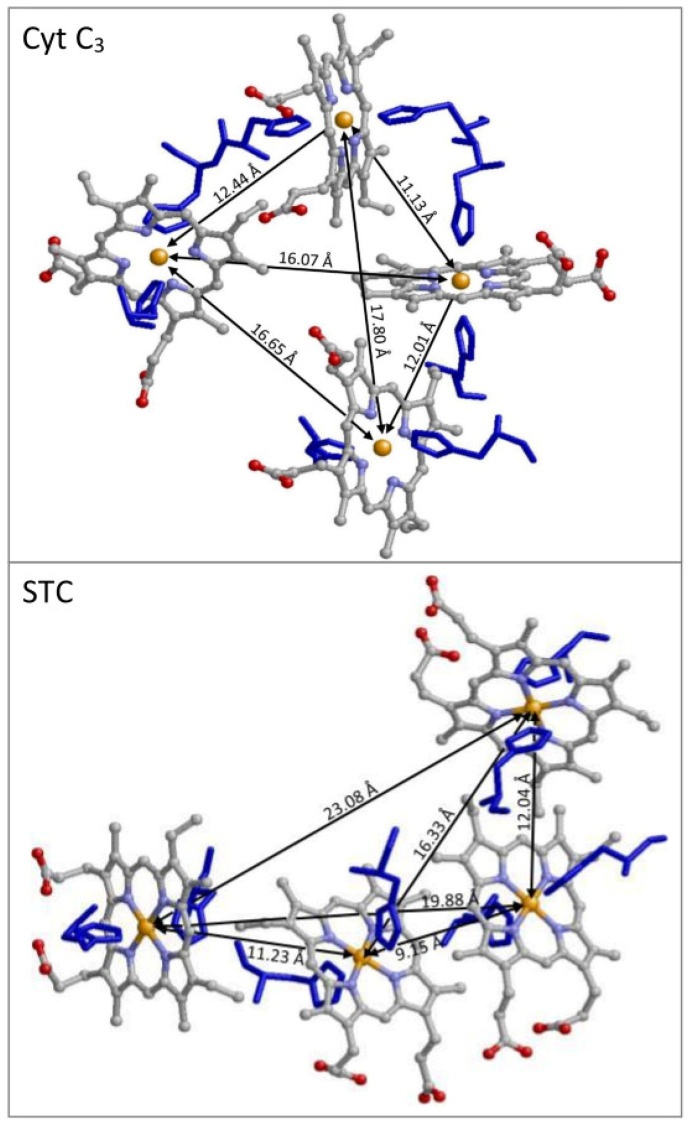
Differential special arrangement of hemes in tetraheme cytochromes. In cytochrome *c*_3_, the four heme irons are at the corners of a distorted tetrahedron, leading to significant dipolar interaction for every pair of Fe-Fe ions. Contrarily, in small tetraheme cytochrome (STC), the heme Fe’s are in a hockey-stick configuration, in which some of the Fe-Fe distances are extremely long. The figure is based on *D. vulgaris c*_3_ (2CTH.pdb [26]) and *S. oneidensis* STC (1M1Q.pdb [27]).

To check whether our previous conclusion on the complication of dipolar interaction in X-band spectra is generally valid, we have now measured the broadband EPR of a different tetraheme cytochrome, with very different spatial arrangement of the hemes (Figure 1). In the previously studied *Desulfovibrio vulgaris* Hildenborough cytochrome *c*_3_, the four iron ions are at the vertices of a deformed tetrahedron [25,26], whereas in the presently studied *Shewanella oneidensis* MR-1 Small Tetraheme Cytochrome (STC), the irons form approximately the pattern of a hockey stick (a ‘dog leg’ according to others [29]), with some of the Fe-Fe distances significantly longer than in the cytochrome *c*_3_ structure [27].

## 2. Results and Discussion

**Figure 2:**
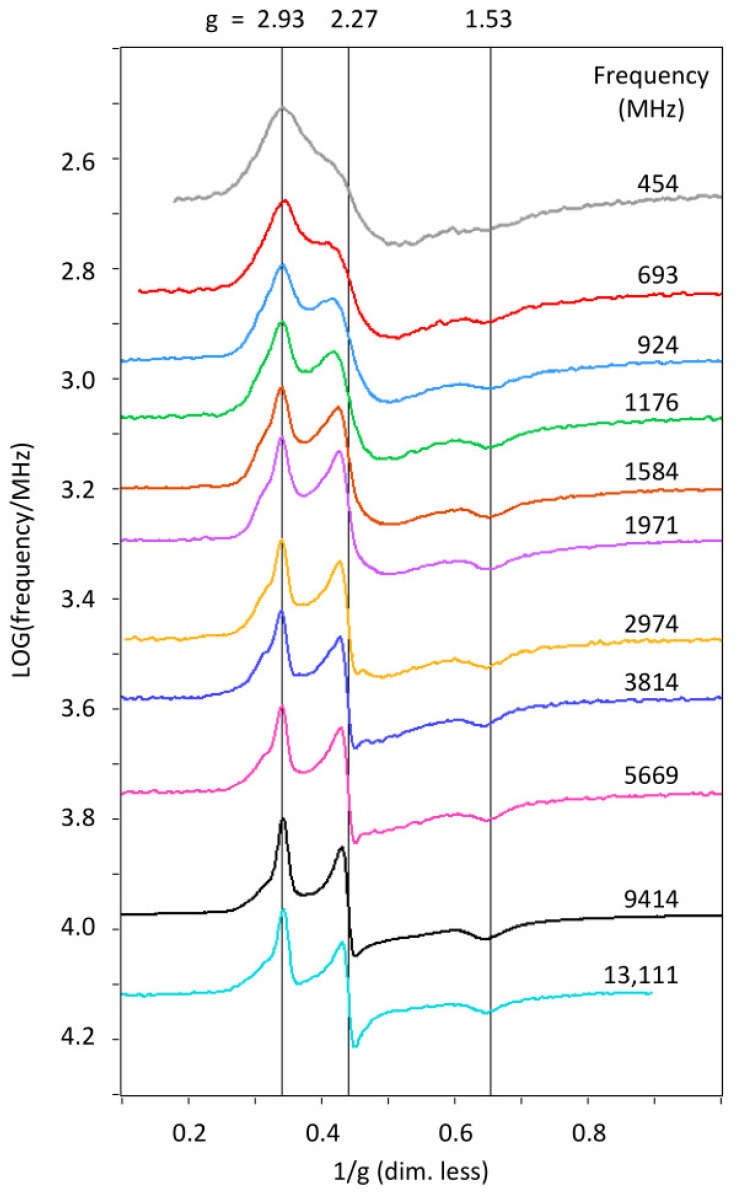
Broadband EPR 2D plot of log (frequency) versus reciprocal *g* value for STC from 454 to 13,111 MHz. The broadening towards low frequency occurs smoothly so that the overall spectral shape is maintained over the full frequency range. Effective *g* values are indicated for the main turning points. The sample is 2 mM *S. oneidensis* STC in 20 mM potassium phosphate buffer, pH 7.6, plus 100 mM KCl. The spectra were recorded with field modulation at 100 kHz and amplitude between 3 and 6 gauss. The temperature was ca. 11 K, and data collection times for individual traces were between 10 and 60 min.

**Figure 3:**
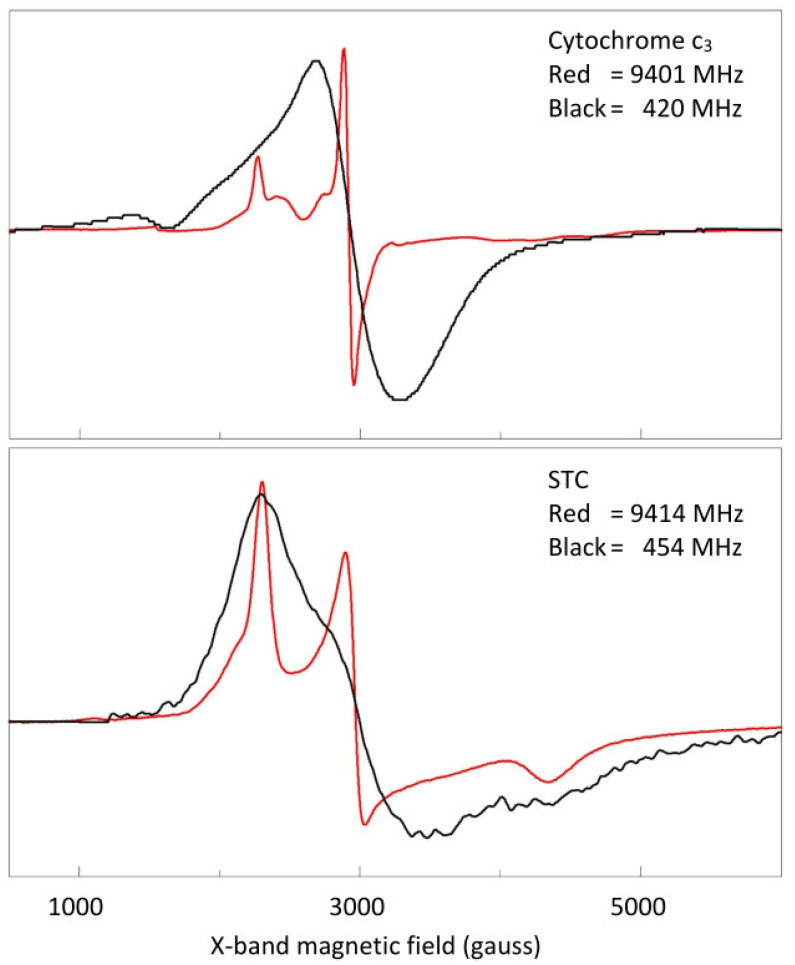
Comparison of the broadening pattern with frequency for cytochrome *c*_3_ and STC. The overlays are to illustrate that a very drastic change in the EPR of *c*_3_ does not have a counterpart in the EPR of STC. In the latter case, the overall spectral shape is retained.

A broadband EPR data set of *S. oneidensis* STC is presented in Figure 2, showing that a sharp spectrum at 13.11 GHz broadens quite smoothly with lowering frequency all the way down to 0.45 GHz, while maintaining its overall pattern of three main features (a positive peak, a derivative shape, and a negative peak) as if all four hemes have similar spectra. This overall broadening pattern is very different from the previously studied *D. vulgaris* cytochrome *c*_3_ [4], in which the broadening came in two waves as a function of reducing frequency, culminating in virtually complete loss of spectral structure at low frequencies. The two different broadening patterns of the two proteins are compared in Figure 3, which shows their X-band spectrum at 9.4 GHz and their low-frequency spectrum at 0.4 GHz on the same reciprocal *g*-value scale. The EPR of *S. oneidensis* STC has not been published before, however, its X-band spectrum (Figure 3) is virtually identical to that reported for STC from *Shewanella frigidimarina* [30,31], whose NMR solution structure [32] shows strong similarity to the crystal structure of *S. oneidensis* STC [27], with a primary sequence identity of 65% between the proteins. From this, we conclude that the spectrum is typical for tetraheme cytochromes with the hockey stick heme location pattern.

The spin Hamiltonian for each individual heme is

(1) $$H=\beta \cdot B\cdot \hat{g}\cdot S+\beta \cdot B\cdot \hat{∆g}\cdot S\;+\sum_{b=1}^{3}\left\{\beta B\left(L\cdot g_{a}\cdot S_{a}+L\cdot g_{b}\cdot S_{b}\right)+\frac{\mu_{0}\beta^{2}}{4\pi r^{3}}\left[\left(g_{a}\cdot S_{a}\right)\cdot \left(g_{b}\cdot S_{b}\right)-\frac{3\left(\overset{⃑}{r}\cdot g_{a}\cdot S_{a}\right)\left(\overset{⃑}{r}\cdot g_{b}\cdot S_{b}\right)}{r^{2}}\right]\right\}\;+\sum_{i}S\cdot {\hat{A}}_{i}\cdot I_{i}$$

in which *β* is the Bohr magneton, *B* is the external magnetic field strength, capped *g* is the *g* matrix, *S* is the electron-spin operator, capped Δ*g* is the *g*-strain matrix, *L* is a unit vector along *B* in the *g*-matrix axes system, *µ_0_* is the permeability of free space, *r* is the length of a vector between the two iron ions under consideration, Fe_a_ and Fe_b_, capped *A* is a hyperfine tensor, and *I* is the nuclear-spin operator. The first term is the electronic Zeeman interaction; the second term is the *g* strain from protein conformational distribution, giving rise to line broadening; the third term is the intramolecular dipolar interaction between hemes, where subscript ‘a’ refers to the heme at hand and subscript ‘b’ is one of the three neighboring hemes; and the fourth term is the superhyperfine (SHF) interaction between the Fe electron spin and the nuclear spin of ^14^N and ^1^H atoms on the porphyrin and the axial His ligands. To further analyze the STC broadband data, we first attempt to simulate its higher-frequency (≥9 GHz) spectra as a sum of four non-interacting hemes, that is, we assume the third and fourth terms in Equation (1) to be negligible. This approach is based on the notion that EPR spectra change their shape with frequency in a broadband data set because they are the result of interactions independent of the frequency and interactions linear in the frequency. In the present case, the magnitude of dipolar interaction is independent of the frequency and so is any hyperfine interaction. On the other hand, the electronic Zeeman interaction, giving rise to the *g* values, is linear in the frequency and so is the broadening mechanism due to conformational distributions, giving rise to *g*-strain broadening. Increasing the frequency will eventually make the dipolar contribution and SHF splittings negligible, leaving a spectrum that is determined only by *g* values and *g*-strain linewidths.

**Figure 4:**
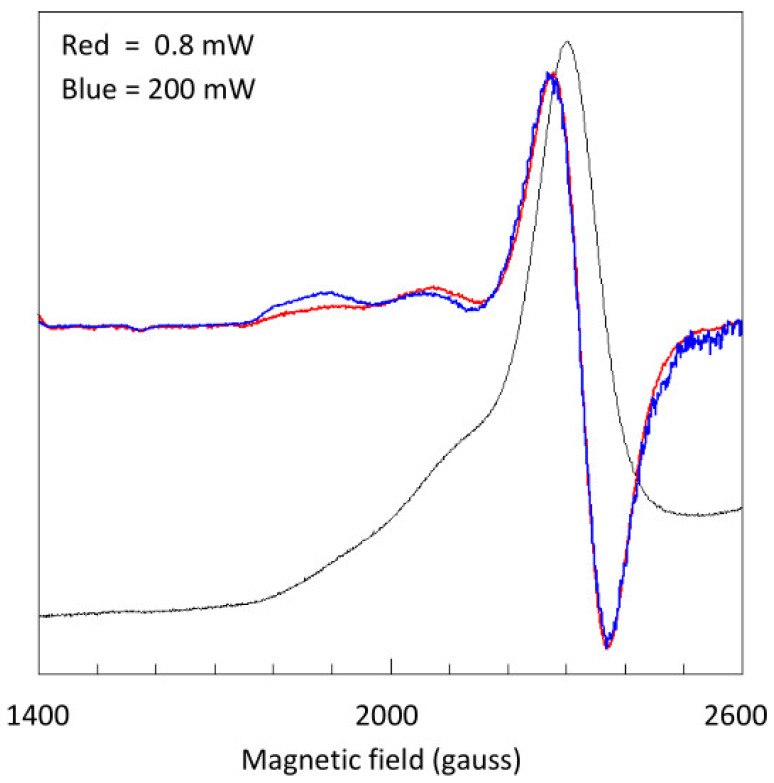
Differential power saturation of two spectral features at the very low-field end of the X-band spectrum of STC. The original black trace has been numerically differentiated to obtain the EPR second derivative as the red trace, and, at maximum power, as the blue trace. Strong relative amplitude changes in these traces show that the shoulders in the original spectrum are *g*_z_ peaks from different hemes. Spectra were taken at 9414.1 MHz, with a modulation amplitude of 5 gauss and a sample temperature of 13 K.

As a preliminary step to a full simulation, we first ask the question of whether the shape of the low-field shoulder in the X-band spectrum is due to two separate *g*_z_ peaks from two hemes or possibly an asymmetric peak from a single heme as a result of non-colinearity of *g*-strain and Zeeman interaction, which is known to afford ‘skewed’ peaks [33]. To address this problem, we try differential saturation, that is, to run this part of the spectrum at widely different microwave power intensities, in an attempt to deconvolute two peaks on the basis of a difference in saturation characteristics. Since these spectral features and their differences are quite small, we emphasize the differences by looking at the EPR second derivative. The result of this inspection is reported in Figure 4: the two shoulders, the peaks in the second-derivative spectrum, clearly differ in saturation behavior, where the second-derivative peak at the lowest field is more difficult to saturate. We conclude that the asymmetric feature is the result of two overlapping *g*_z_ peaks from two different hemes.

**Figure 5:**
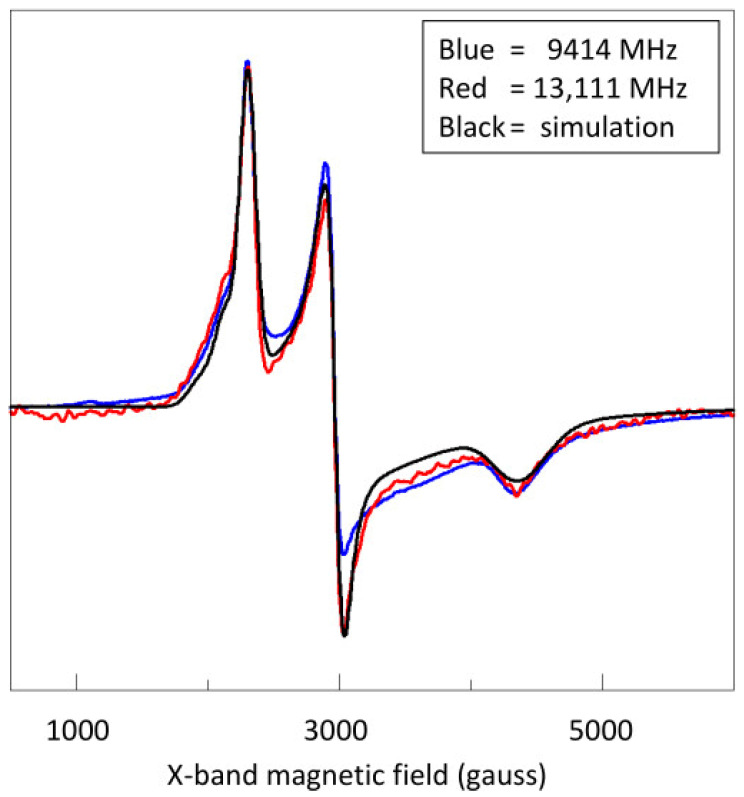
Simulation of the 13.1 GHz spectrum of *S. oneidensis* STC assuming four low-spin hemes of equal concentration, with the parameters given in Table 1. Comparison with the spectrum at 9.4 GHz indicates that dipolar interaction is significant at X-band.

We now simulate the spectrum at 13.1 GHz as a stoichiometric sum of four hemes, and we compare it with the experimental spectra at 13.1 and 9.4 GHz (Figure 5). The simulation parameters are given in Table 1. As is common for this type of spectra, those with the highest *g*-anisotropy have *g*_z_ peaks of relatively low amplitude and *g*_y_, and especially *g*_x_, features that are broad, and therefore, they are difficult to identify by visual inspection. To limit the fitting parameter space, the *g* values are constrained to obey the theoretical relation [34]

(2) $$\sum_{i}g_{i}^{2}\approx 16$$

which has been suggested to be a fair approximation for hemes with 3 ≤ *g*_z_ < 4 [35]. The simulation gives a reasonable fit to the 13.1 GHz spectrum, but there are some misfits to the 9.4 GHz spectrum, most notably in the negative lobe of the *g*_y_ feature. This observation implies that the X-band spectrum is complicated by a small but significant contribution from dipolar broadening, and thus, a simulation of that spectrum assuming Zeeman interaction plus *g*-strain only would not necessarily result in a correct determination of *g* values.

**Figure 6:**
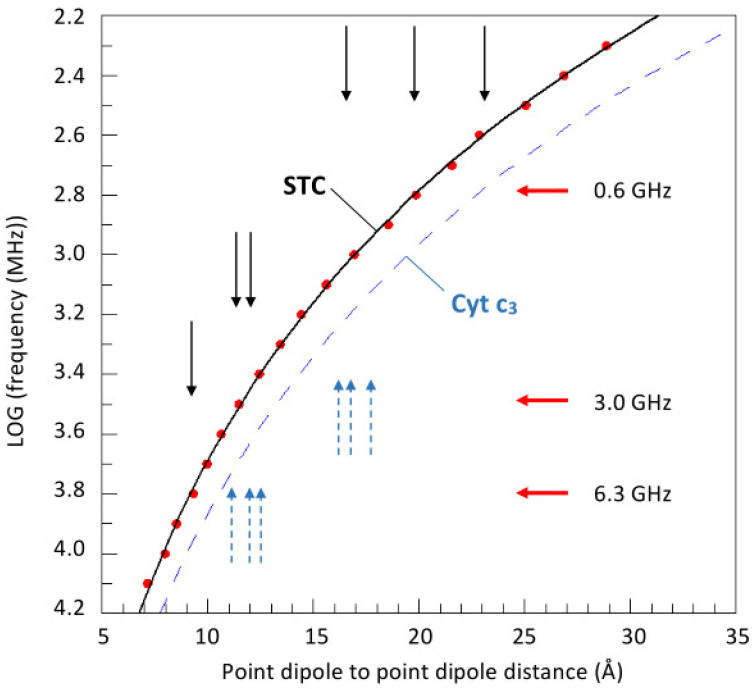
Semi-quantitative identification of the onset of broadening as a function of frequency in the EPR of *S. oneidensis* STC and comparison with a previous analysis for *D. vulgaris c*_3_ [4]. The heme with the narrowest line width from *g* strain (component 1 in Table 1) was taken as an approximate monitor of the very onset of broadening under the point-dipole model, when the Fe-Fe distance dipolar interaction with an identical center would cause the sharp derivative feature at *g*_y_ = 2.26 to be broadened by 20%. The Fe-Fe distances in STC are indicated by the black arrows and those in *c*_3_ by the broken blue arrows. Grouping of the arrows suggests the occurrence of ‘waves’ of broadening below the indicated frequencies, although the sorting of Fe-Fe distances in STC affords a rather diffuse pattern, in contrast to the sorting into two well-separated groups in cytochrome *c*_3_.

To put this conclusion on a more quantitative footing, we adopt the procedure previously used for the analysis of broadband EPR from tetraheme cytochrome *c*_3_ [4]. From Table 1, we take the heme spectrum with the sharpest linewidth and we ask the semi-quantitative question: what would be the dipolar interaction between two co-linear hemes of this sharpness as a function of iron-iron distance and microwave frequency? As a criterion for observable broadening, we take a 20% increase in apparent linewidth of the sharpest feature of the spectrum, which is the *g*_y_ derivative-shaped feature. The answer should provide an approximate frequency below which we expect to start to observe dipolar broadening. The analysis is illustrated in Figure 6, in which the calculated red points should provide a frequency limit above which broadening in STC is not likely to be observable. Since three out of four spectra are actually broader than the sharpest one, in reality, the onset of broadening for a given Fe-Fe distance should only be observable at lower frequencies, that is, in the area above the black line connecting the calculated points. A broken blue trace is also given in Figure 6 as previously calculated for cytochrome *c*_3_. That line is shifted to the right because the EPR spectrum of cytochrome *c*_3_ has features that are sharper than those of the STC spectrum (cf. Figure 3). The vertical black arrows correspond to the six Fe-Fe distances in STC. When we arrange these into three groups as one short distance (9.15 Å), two intermediate ones (on average 11.6 Å), and three long ones (rather widely distributed around circa 20 Å), we then obtain three limiting frequency values, below which we should observe three ‘waves’ of broadening. In the broadband EPR of cytochrome *c*_3_, two waves were observed [4], due to the sorting of six Fe-Fe distances into two well-defined groups. However, with STC, their identification is less obvious in the spectra of Figure 2. One reason is that in the hockey-stick arrangement, three of the Fe-Fe distances are apparently too long to afford significant dipolar effects. Also, the combination of relatively broad spectral components, compared to those of *c*_3_ and the other three Fe-Fe distances being of a quite similar length, apparently results in a rather phaseless, diffuse increase in broadening with decreasing frequency.

**Figure 7:**
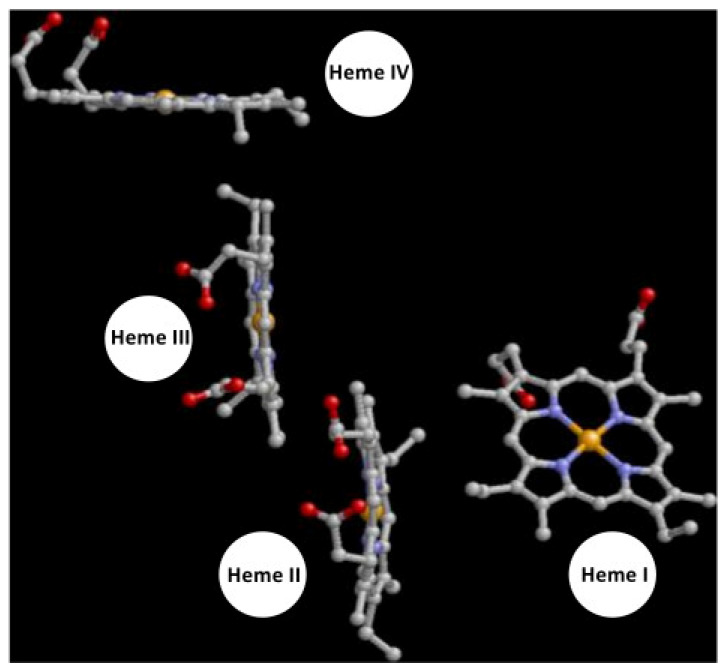
View of the hemes in *S. oneidensis* STC from an angle to emphasize the relative orientation of the tetrapyrrole macrocycles. The two central hemes are parallel, whereas the peripheral hemes are perpendicular to each other, and to the central pair. Heme numbering is according to the order of covalent Cys bonding in the protein sequence. Figure based on 1M1Q.pdb [27].

Figure 7 presents a view of the hemes from an angle to emphasize that two of the hemes (the two central ones) have their tetrapyrrole rings in essentially parallel orientation. The Roman numbering follows the heme order attachment to the polypeptide chain via thioether links to eight cysteine residues [31]. The distance between these two hemes is short 9.15 Å. The other two hemes, the peripheral ones, have their tetrapyrrole ring at a large angle from the central hemes. The first onset of broadening, observed between 13.1 and 9.4 GHz, is not associated with the interaction between the two hemes giving rise to the two sharp spectra (hemes 1 and 2 in Table 1): at least a part of the sharp *g*_y_ feature is not broadened over this frequency range although another part of the derivative-shaped feature is broadened, suggesting that a broad spectrum should be assigned to one of the central hemes and a sharp spectrum to the other.

The next two shortest Fe-Fe distances are from each of the central hemes to their neighboring peripheral heme. Figure 6 predicts that the onset of the second wave of broadening occurs around 3 GHz, and indeed we see that the remaining sharp *g*_y_ feature starts to broaden, which is consistent with one of the peripheral hemes being associated with a sharp spectrum. Importantly, the coincidence of the predicted and observed frequency threshold suggests that any deviation from the point-dipole model of interaction should be small in contrast to the previous findings with intermolecular dipolar interaction in cytochrome *c* [4]. The remaining three long Fe-Fe distances do not group closely, and indeed, in the lower frequency range, we do not see any clear ‘wave’ or breakpoint. The broadening increases smoothly with decreasing frequency, which is also consistent with the predictions of Figure 6 based on the point-dipole model.

In a previous NMR study on the *S. frigidimarina* STC, a calculation was attempted of *g* values (reproduced in Table 1) of the four hemes based on magnetic susceptibility tensor values deduced from ^13^C shifts [31]. Qualitatively, the calculated *g* values correspond reasonably well with the sets determined here in the simulation of the 13 GHz spectrum. Some discrepancies, however, are notable. For example, the calculated value (*g*_z_ = 3.61) of Heme II is at the extreme limit of the experimental EPR spectrum, and it has an amplitude too low for a system with 1:1:1:1 stoichiometry. Also, the sum of the squares of the *g* values for this heme amounts to 15.3, which falls rather significantly short of the predicted one of 16 to hold, specifically for the hemes with *g*_z_ values approaching 4 [35]. Perhaps more consequential is the exchange of a broad and sharp spectrum for hemes III and IV, compared to the present analysis. Here, *g*_z_ = 3.21 calculated for heme IV is not found in the experimental spectrum (its position falls in between the two *g*_z_ peaks detected in Figure 4) and also *g*_x_ = 1.38 for heme III appears to be missing experimentally, which makes the assignment of *g* matrices to the hemes III and IV less convincing.

Our attempts to assign individual spectra to individual hemes thus far are far from complete. We will, therefore, fully use the available crystallographic structure information to extend the assignment. There are 24 possible configurations in total to assign each of the four heme spectra (*g* matrices and *g*-strain parameters of heme 1, 2, 3, and 4 in Table 1) to each of the four hemes in the protein structure (heme I, II, III, and IV in Figure 7). For each configuration, we calculate the sum of the four EPR spectra, and each spectrum is broadened by the dipolar interaction with the other three hemes. From the X-ray structure, we obtain not only the heme-heme distances but also the relative orientations of the hemes. We assume that the latter define the relative orientations of the *g* tensors, which we obtain as Euler rotation angles between the axis systems defined through the central iron atom, the N1 and N2 atoms of the tetrapyrrole ring, and the εN of the first coordinating His ligand in the protein sequence. These rotation angles allow us to compute the rotated *g* tensor of each of the three hemes with respect to the heme for which we calculate the spectrum subject to dipolar broadening according to Equation (1). Possible breakdown of the point-dipole model is introduced as a dipolar sphere around the Fe point by adding a reduction factor to the Fe-Fe distances equal to the sphere’s diameter. Superhyperfine (SHF) interaction with neighboring ^14^N and ^1^H nuclei is also included, where initial simulations make it clear that, at lower frequencies, SHF should be included as an unresolved envelope of splittings to account for the low resolution of splittings from dipolar interactions. We encountered this situation before in the analysis of broadband EPR from cytochrome *c*_3_, where the SHF was modeled as a Gaussian envelope, whose width was deduced from the individual splittings reported in ENDOR and ESEEM studies. Due to a lack of angle-dependent data in the latter studies, the width is assumed to be isotropic [4].

**Figure 8:**
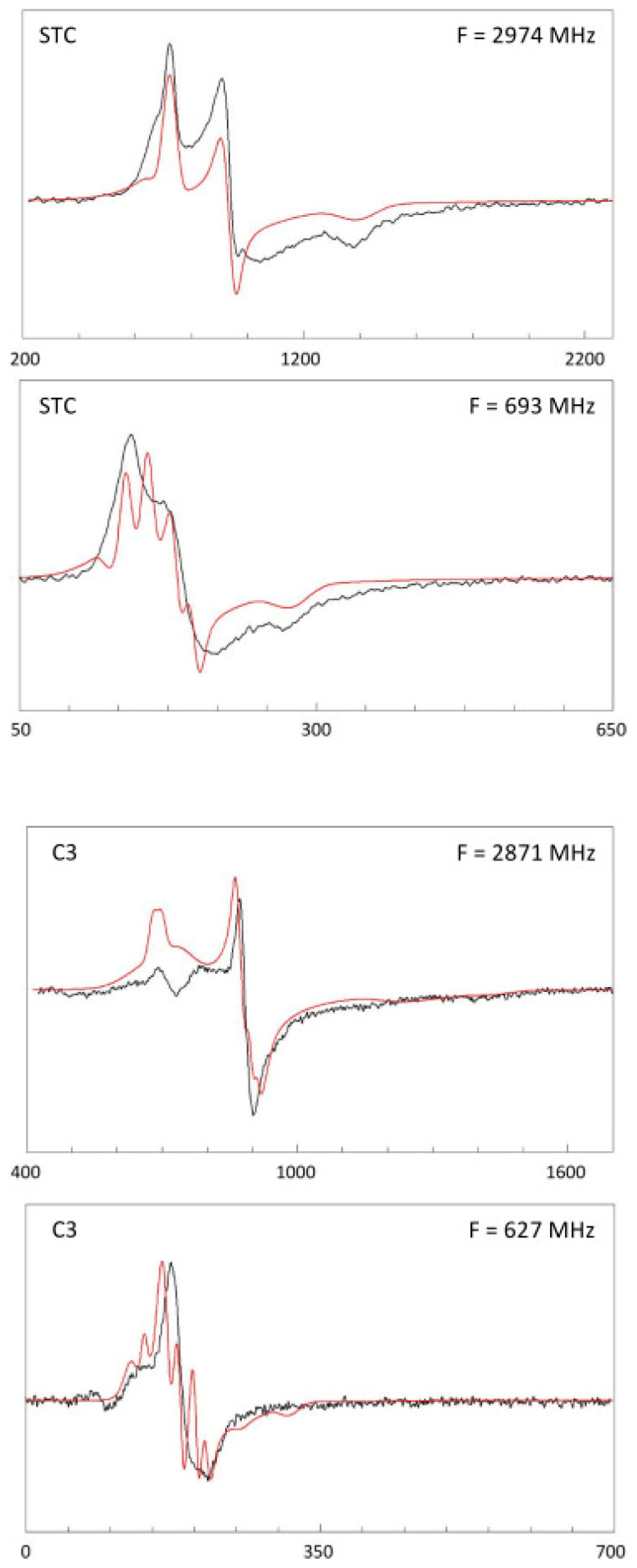
Simulations based on Equation (1) of STC and *c*_3_ EPR at intermediate (3 GHz) and low (0.7 GHz) frequencies. The simulations are for one particular assignment out of 24 possibilities of the four spectral components (Table 1) to the four hemes (Figure 7). These individual fits are for assignments 1-3-4-2 (STC) and 1-4-2-3 (*c*_3_). An overview of the simulations for all 24 configurations can be found in Figures S1–S4.

Figure 8 gives an example of the simulations of the *S. oneidensis* STC spectrum in comparison with that of the *D. vulgaris* cytochrome *c*_3_ spectrum at intermediate (ca. 3 GHz) and low (ca. 0.7 GHz) microwave frequencies, based on the previously determined values for the *g* matrix and *g* strain at ca. 13 GHz (Figure 5) with the dipolar interaction pinned down to the 3D structure of the protein, and with additional broadening by unresolved superhyperfine splittings from tetrapyrrole and histidine N and porphyrin H atoms [4]. The figure is for a single configuration, that is, for a single assignment of the spectroscopic components 1–4 from Table 1 to the four hemes I–IV. The complete data sets for all 24 configurations can be found in Figures S1 and S2. Despite the sophisticated nature of the model, the simulations in Figure 8 are disappointingly inaccurate fits. On the other hand, many of the other 24 configurations give worse fits (Figures S1 and S2). The ‘best’ or ‘least worse’ of the 24 fits are any of the combinations of the spectral components 1-3-4-2, 1-4-3-2, 3-2-4-1, or 3-4-1-2 in Table 1 to the actual hemes I-II-III-IV, with the counting order of the latter determined by their covalently bonding through Cys residues to the polypeptide chain. The four ‘best’ fits are essentially equal, however, we have previously argued (in relation to Figure 7) that one of the central hemes and one of the peripheral hemes should have a sharp spectrum, which would reduce the set to the combinations 3-2-4-1 or 3-4-1-2. Similarly, for *D. vulgaris c*_3_, we identify the configurations 1-2-4-3, 1-4-2-3, 4-2-3-1, and 4-3-2-1 to be assigned to the hemes I-II-III-IV (Figure 8, Figures S3 and S4), but since three out of four spectra are sharp and there is no standing out shortest distance, all four configurations remain equally likely.

Why do the ‘best’ fits of Figure 8 fall significantly short of what we would normally consider to be ‘good’ fits? First, the *g* values and strain broadenings were determined at 13 GHz only, assuming colinearity of the *g* matrix and the *g*-strain tensor. Non-colinearity can give rise to asymmetries in the spectral features [33], which we have ignored here to keep the simulation problem tractable. Second, we have limited the set of *g*_xyz_ values to approximately obey Equation (2), which is essentially based on the assumption of ionic coordination [34]. Third, since the *g* values are distributed due to a distribution of protein conformations, the mutual Fe-Fe dipolar interactions may also be well distributed, leading to distributions in Fe-Fe distances and in the Euler angles between the *g* matrices, which we have also ignored for tractability reasons. Fourth, for a lack of data on STC and c_3_, the unresolved superhyperfine splittings were deduced from the ENDOR and ESEEM values of other hemoproteins, and these data themselves are rather incomplete in terms of anisotropy [4].

We have also assessed the effect of correcting the point-dipole model by introducing a dipolar sphere around each Fe, which comes down to an effective reduction of the Fe-Fe distances. In all the mid- and low-frequency simulations, both for STC and *c*_3_, this led to deterioration of the fit, namely, greater splittings, which would fall outside the experimental spectral envelope (not shown). We conclude that deviations for the point dipole model (at least for intramolecular interactions in tetraheme cytochromes) are not indicated, which is in contradiction to a previous suggestion [4].

## 3. Materials and Methods

The STC from *Shewanella oneidensis* MR-1 was isolated and purified as described previously [24].

**Figure 9:**
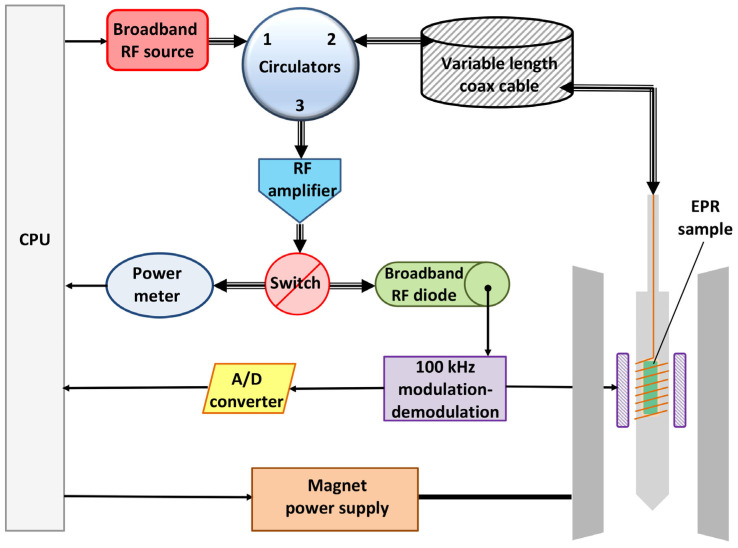
Schematic overview of the broadband EPR spectrometer. See the text for an explanation. The figure was taken from Ref. [4].

The broadband EPR spectrometer, its cryogenics, and its dedicated software have been described in detail in [4,5,6,7,8]. In brief (cf. Figure 9), the setup uses a conventional scanning electromagnet and conventional 100 kHz magnetic-field modulation with two coils to the sides of the sample. The non-conventional part starts with a broadband (DC-18 GHz) tunable monochromatic source sending microwaves into a set of circulators, from where the waves continue via a length-adjustable coaxial cable (for mode adjustment) into the resonator/sample holder. The latter is the most unconventional part of the setup: it is not a classical single-mode cavity, but a wire microstrip structure that can be made resonant over a large number of frequencies. After absorption by the sample, the attenuated wave is reflected back into the circulator to be routed into a detection system either to identify the resonant mode with a power meter as a function of frequency or to detect the EPR via a broadband (DC-18 GHz) detector diode connected to the signal demodulation electronics. Additionally, data in X-band were collected with a Bruker EMXplus spectrometer (Bruker Physik AG, Karlsruhe, Germany).

New software specific for tetraheme EPR analysis, using structural information from pdb files, was written in LabVIEW2020 and Intel FORTRAN. The use of the software is described in detail in Supplementary Material, and the source code and executables are provided in a separate zip folder “Software for tetraheme analysis.zip”. In brief, the *g* values and *g*-strain linewidths are determined for the arbitrarily labeled hemes 1, 2, 3, and 4 in a simulation of a high-frequency spectrum. Then, from crystallographic information we label the hemes as I, II, III, and IV according to their covalent-binding order to the protein sequence, and we calculate the Fe-Fe vectors and Euler angles of relative heme-heme orientations. From the latter, the rotations of the *g* matrices are computed for three hemes with respect to a reference heme. Finally, for all 24 permutation assignments of hemes 1, 2, 3, and 4 (g values) to hemes I, II, III, and IV (structural data), we compute the EPR spectra subject to dipolar interaction between all hemes at low microwave frequencies, and we compare the results to experimental spectra.

## 4. Conclusions

The primary goal of the present study was to establish how heme-heme dipolar interactions materialize in low-frequency broadband EPR spectroscopy, to what level of accuracy these interactions can be numerically analyzed and subsequently interpreted, and what weaknesses in the procedure of analysis remain to be addressed in future work. The analysis of broadband EPR data from tetraheme cytochromes by making use of 3D structural data (distances between Fe ions and Euler angles between Fe-N(heme)-N(heme)-N(His) axes systems) allows for the assignment of individual EPR spectral components to actual hemes, with an elimination of the majority of the 24 theoretically possible assignments. The lack of quality of individual fits suggests that we still do not fully understand all the details of these complex spectra and it may be advisable to re-visit and re-activate the slumbering research field of tetra-heme EPR spectroscopy. Further support for this conclusion is found in the observation of dipolar effects in the X-band spectra of these proteins, which is not only unpredicted (e.g., by the analysis in Figure 6) but also implies that exact *g* values cannot be obtained from X-band analysis on the assumption of dipolarly non-interacting hemes.

In order to obtain better individual spectral component fits and to uniquely assign these components to specific hemes, the present analysis may be improved in the future by extending the data set to include spectra taken at intermediate potentials in redox titrations. The extent of such an experimental and theoretical effort is not to be underestimated. An early X-band EPR titration study of *D. vulgaris* cytochrome *c*_3_ at pH 8.0 suggested that individual heme reduction potential values were mutually close, which excludes the experimental quantitative separation of individual spectra [36]. Note also that the *g*_z_ values (based on visual inspection and ignoring dipolar interaction) reported in that study differ quite significantly from those based on spectral simulations in Table 1. Direct electrochemistry at pH 7.0 indicates that the highest *E*_m_’ value becomes better separated from the other three at this pH [17]. These early observations suggest that a meaningful extension of our present data set would require taking multiple data points in redox titrations at several pH values, submitting each data point to broadband EPR spectroscopy, and somehow globally analyzing all these data in terms of the spin Hamiltonian in Equation (1) and the 24 configurations described above. As a technical annex, one would have to develop a procedure, and possibly with hardware, to allow for the anaerobic transfer of titration samples to the broadband resonator/sample holder and its anaerobic cooling to cryogenic temperatures.

What is the biochemical relevance of the present study? The broadband EPR spectra from cytochrome *c*_3_ (with four hemes in a distorted tetrahedral arrangement) are quite different from those from STC (with four hemes in a hockey-stick arrangement). The former show a more pronounced variation with microwave frequency, presumably related to the shortness of all six Fe-Fe distances. But the *c*_3_ spectra are also significantly sharper than the STC ones, attesting to the fact that the *g* strain in *c*_3_ is reduced compared to that in STC. *g* Strain is a reflection of protein conformational distribution, and the *g*-strain width is a measure of the flexibility of a protein or the lack thereof [37]. Thus, the EPR data indicate that *c*_3_ has a more rigorous 3D structure than STC, which may well be related to the biological function of *c*_3_ as an effective two-electron transfer protein to electron-pair reactions catalyzing enzymes. The behavior of 2e^−^ donor/acceptor is the result of pronounced redox-Bohr interactions between the hemes of *c*_3_ [38,39,40]. On the contrary, the EPR of STC suggests that the protein is more flexible and perhaps subject to less consequential redox interactions, which would be congruent with the proposal that it rather acts as a ‘linear electron wire’ [41] or perhaps even as an indiscriminate ‘electron harvesting’ protein [27]. This differential flexibility may be related to differences in structural motifs, such as the twice occurrence of a CXXXXCH pattern in *c*_3_ (instead of the classical CXXCH pattern exclusively in STC) and the consecutiveness in the sequence of two axial ligands to two different hemes in *c*_3_ [25]. On the other hand, the parallel orientation of hemes, present in STC and absent in *c*_3_, does not correlate with the electron-pair redox behavior.

In summary, we think that we have made a notable step in advancing the measurement, analysis, and interpretation of dipolar interaction in multiheme cytochromes. At the same time, there is still significant room for future procedural improvement. In terms of biochemical relevance, the differences in dipolar broadening between cytochrome *c_3_* versus the STC protein appear to correspond to the differences in their biological functions in electron transfer.

## Figures and Tables

**Table 1:** *g* Values and linewidth of the hemes in STC and *c*_3_. (A) parameters obtained from fitting the 13.11 GHz spectrum of *S. oneidensis* STC, assuming broadening by co-linear *g* strain only, and assuming the sum of the squares of the *g* values to be approximately equal to 16. (B) reproduction of the previously reported *g* values calculated from the NMR data of *S. frigidimarina* STC [31]. (C) reproduction of the previous fit to the X-band EPR of *D. vulgaris* cytochrome *c*_3_ [4].

**(A)**		**Heme 1**		**Heme 2**		**Heme 3**		**Heme 4**
*g*_z_		2.94		2.895		3.11		3.33
*g*_y_		2.26		2.28		2.1		1.95
*g*_x_		1.52		1.53		1.4		1.1
∑*g*_i_^2^		16.06		15.92		16.04		16.1
*W*_z_^b)^		0.09		0.08		0.14		0.25
*W*_y_		0.042		0.07		0.22		0.35
*W*_x_		0.08		0.08		0.3		0.3
**(B)**		**Heme I**		**Heme II**		**Heme III**		**Heme IV**
*g*_z_		2.89		3.61		2.99		3.21
*g*_y_		2.31		1.48		2.26		2.1
*g*_x_		1.49		0.28		1.38		1.1
∑*g*_i_^2^		15.91		15.3		15.95		15.92
**(C)**		**Heme 1**		**Heme 2**		**Heme 3**		**Heme 4**
*g*_z_		2.76		2.94		2.963		3.15
*g*_y_		2.318		2.3		2.3		2.27
*g*_x_		1.665		1.555		1.438		1.1
*W*_z_		0.155		0.14		0.042		0.16
*W*_y_		0.018		0.018		0.018		0.024
*W*_x_		0.075		0.055		0.048		0.125

## Data Availability

The data are contained within the article.

## Supplementary Materials

The following supporting information can be downloaded at: https://www.mdpi.com/article/10.3390/ijms241612713/s1.
