# Supplementary material for: A Comparative Multi-Frequency EPR Study of Dipolar Interaction in Tetra-Heme Cytochromes
Source: Int J Mol Sci. 2023 Aug 12;24(16):12713. doi: 10.3390/ijms241612713 (PMC10454114; doi:10.3390/ijms241612713)
Supplement: Supplementary file 1 [file ijms-24-12713-s001.zip › supplementary.pdf]

Supplementary Material with

A comparative multi-frequency EPR study of dipolar interaction in tetra-heme cytochromes

By Wilfred R. Hagen and Ricardo O. Louro

Figures S1–S4. Simulations, based on Equation 1, of EPR from *S. oneidensis* small tetraheme cytochrome, STC, and *D. vulgaris* cytochrome c_3 at intermediate (3 GHz) and low (0.7 GHz) frequency. The simulations take into account crystallographic structural information on the Fe-Fe distances and the relative orientation of the hemes. Superhyperfine interaction from ^{14}N and ^{1}H nuclei is accounted for by convolution with a Gaussian line of 3.91 gauss width (HWHH). The simulations cover all 24 possible assignments of the four spectral components (Table 1) to the four hemes (Figure 7). Figure S1 is for STC at 2974 MHz; Figure S2 is for STC at 639 MHz; Figure S3 is for c_3 at 2871 MHz; Figure S4 is for c_3 at 627 MHz.

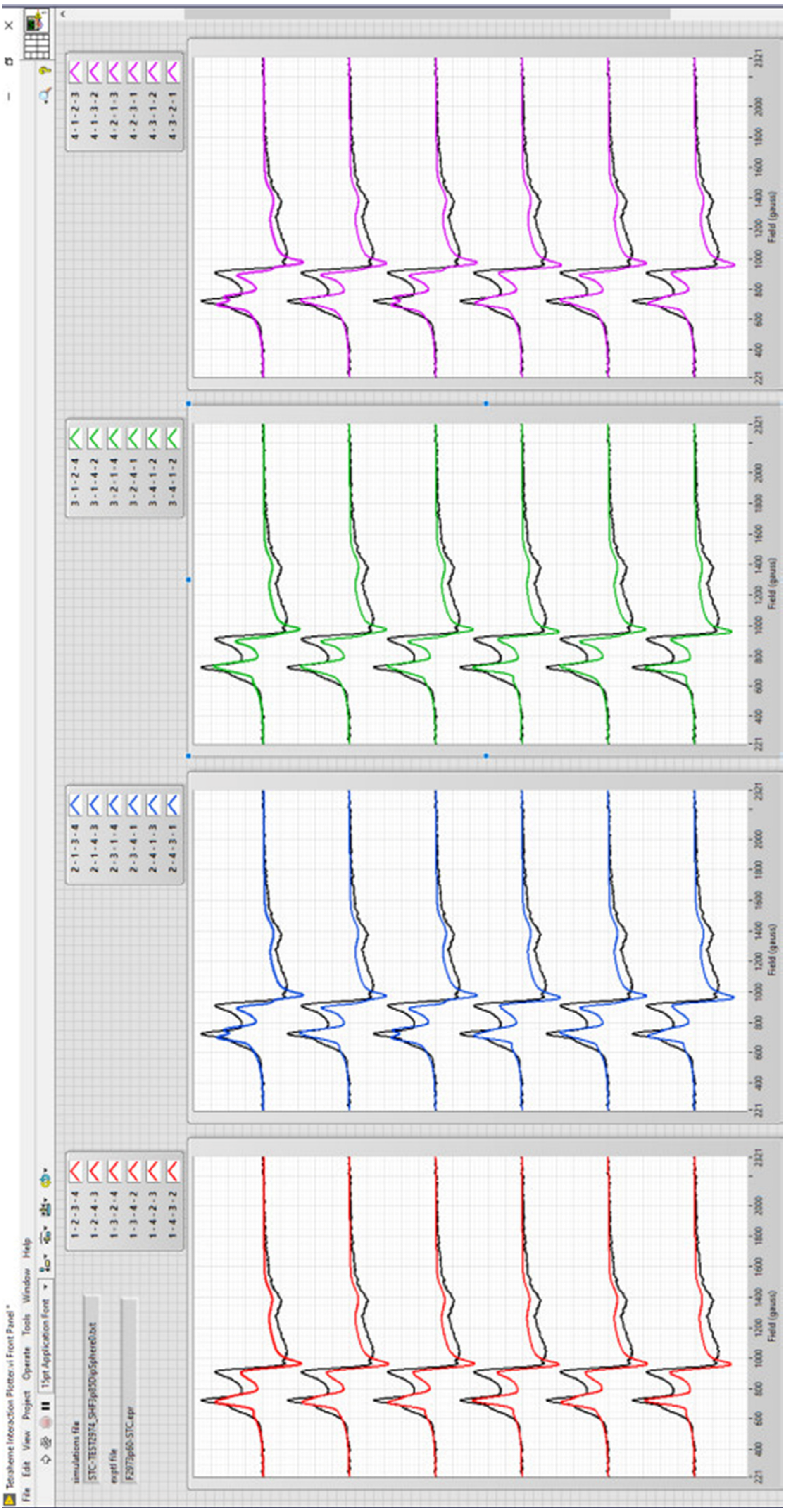
simulations file
exptl file
1 - 2 - 3 - 4
1 - 2 - 4 - 3
1 - 3 - 2 - 4
1 - 3 - 4 - 2
1 - 4 - 2 - 3
1 - 4 - 3 - 2
2 - 1 - 3 - 4
2 - 1 - 4 - 3
2 - 3 - 1 - 4
2 - 3 - 4 - 1
2 - 4 - 1 - 3
2 - 4 - 3 - 1
3 - 1 - 2 - 4
3 - 1 - 4 - 2
3 - 2 - 1 - 4
3 - 2 - 4 - 1
3 - 4 - 1 - 2
3 - 4 - 1 - 2
4 - 1 - 2 - 3
4 - 1 - 3 - 2
4 - 2 - 1 - 3
4 - 2 - 3 - 1
4 - 3 - 1 - 2
4 - 3 - 2 - 1
Field (gauss)
221
400
600
800
1000
1200
1400
1600
1800
2000
2321

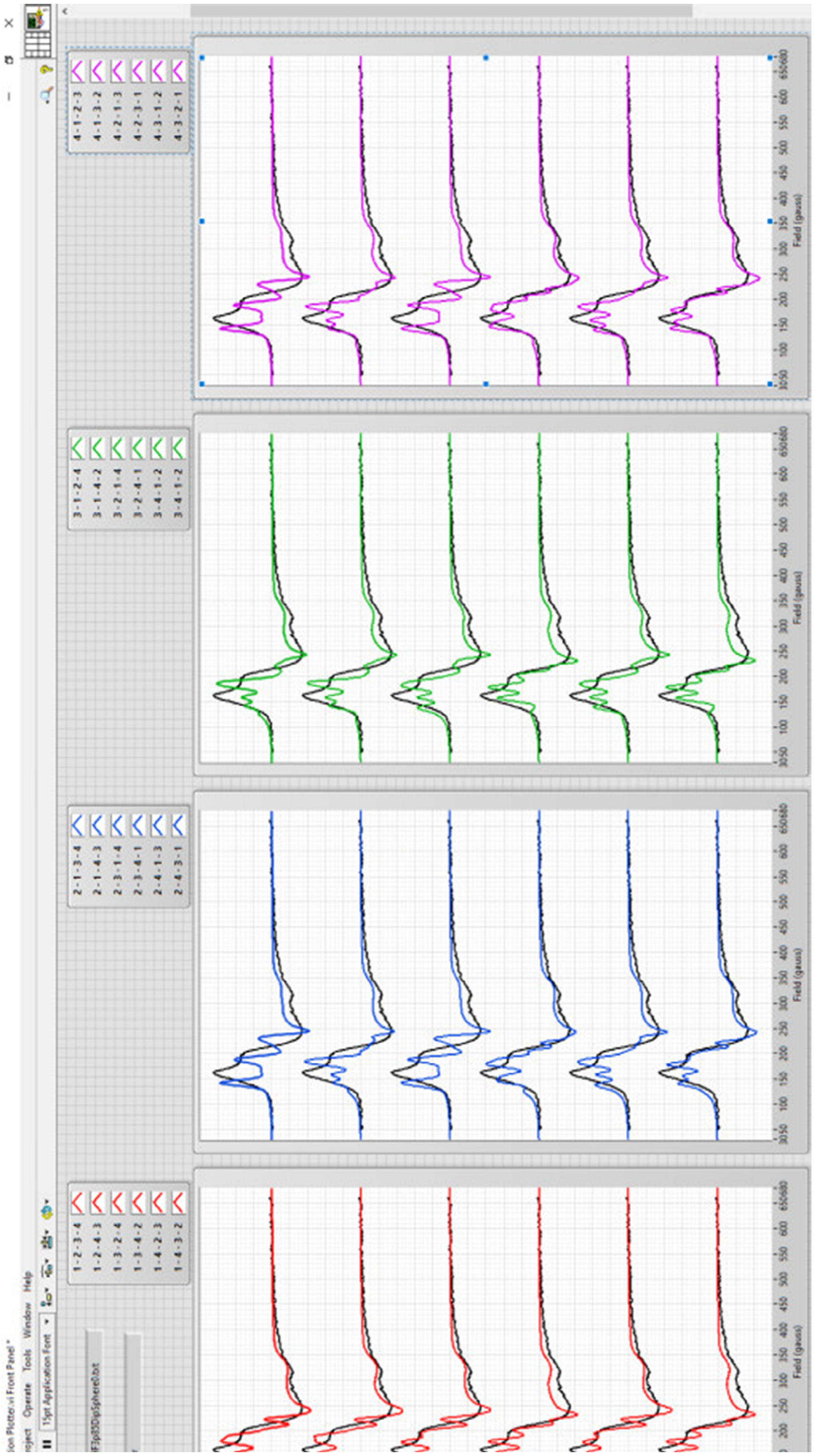
1-2-3-4
1-2-4-3
1-3-2-4
1-3-4-2
1-4-2-3
1-4-3-2
2-1-3-4
2-1-4-3
2-3-1-4
2-3-4-1
2-4-1-3
2-4-3-1
3-1-2-4
3-1-4-2
3-2-1-4
3-2-4-1
3-4-1-2
3-4-2-1
4-1-2-3
4-1-3-2
4-2-1-3
4-2-3-1
4-3-1-2
4-3-2-1
Field (gauss)
